# Gut Micro- and Mycobiota in Preeclampsia: Bacterial Composition Differences Suggest Role in Pathophysiology

**DOI:** 10.3390/biom13020346

**Published:** 2023-02-10

**Authors:** Sofie Meijer, Elena Pasquinelli, Sonia Renzi, Shahram Lavasani, Mehrnaz Nouri, Lena Erlandsson, Duccio Cavalieri, Stefan R. Hansson

**Affiliations:** 1 Division of Obstetrics and Gynecology, Department of Clinical Sciences Lund, Lund University, 22185 Lund, Sweden; 2 Department of Obstetrics and Gynecology, Skåne University Hospital, 22242 Lund, Sweden; 3 Medical Genetics, University of Siena, 53100 Siena, Italy; 4 Med Biotech Hub and Competence Center, Department of Medical Biotechnologies, University of Siena, 53100 Siena, Italy; 5 Department of Biology, University of Florence, 50121 Florence, Italy; 6 Department of Biology, Lund University, 22362 Lund, Sweden; 7 ImmuneBiotech AB, Medicon Village, 22381 Lund, Sweden

**Keywords:** preeclampsia, microbiome, mycobiome, calprotectin

## Abstract

Preeclampsia is a severe pregnancy-related inflammatory disease without an effective treatment. The pathophysiology remains partly unknown. However, an increased inflammatory response and oxidative stress are part of the maternal systemic reaction. Recent data have suggested that dysbiosis of the gut microbiome plays a role in preeclampsia as well as other inflammatory diseases. However, dysbiosis in preeclampsia has not been studied in a Scandinavian population. Furthermore, although the fungal flora may also have anti-inflammatory properties, it has never been studied in preeclampsia. We included 25 preeclamptic and 29 healthy third-trimester women for the ITS and 16S sequencing of fungal and bacterial microbiota, respectively. Calprotectin was measured to assess systemic and intestinal inflammatory responses. The fungal diversity differed with BMI and gestational length, suggesting a link between fungi and the immune changes seen in pregnancy. An LEfSe analysis showed 18 significantly differentially abundant bacterial taxa in PE, including enriched *Bacteroidetes* and depleted *Verrucomicrobia* and *Syntergistota* at the phylum level and depleted *Akkermansia* at the genus level, suggesting a role in the pathophysiology of PE.

## 1. Introduction

Preeclampsia (PE) is a leading cause of maternal and fetal morbidity and mortality worldwide. To date, the only cure is to deliver the baby and thereby the placenta [1].

PE is a pregnancy-specific condition characterized by gestational hypertension in combination with proteinuria and/or other manifestations of maternal organ dysfunction, including fetal growth restriction, arising after 20 weeks of pregnancy [2]. Globally, 3–7% of all pregnancies are affected, which constitutes a total of 8.5 million women every year [1]. A range of short- and long-term complications can follow for both the mother and the child, including an increased long-term risk of cardiovascular disease [3,4]. Risk factors for developing PE include nulliparity, advanced age of the mother (≥40 years), obesity, diabetes, hypertension, renal disease, the presence of antiphospholipid antibodies, in vitro fertilization, and a family history of PE [2]. Many of these risk factors overlap with risk factors for cardiovascular disease.

The underlying etiology behind PE is still largely unknown. However, placental stress is central to the pathophysiology, with different etiologies in early-onset (<34 gestational weeks) and late-onset PE (≥34 gestational weeks) [5]. Early-onset PE is mainly caused by defective placentation due to a shallow migration of cytotrophoblasts into decidual spiral arteries, resulting in defective vascular remodeling [5,6]. This gives rise to hypoperfusion, ischemia, oxidative stress, and tissue damage in the placenta. In late-onset PE, placental stress is likely caused by a combination of senescence of the placenta and a maternal genetic predisposition to metabolic and cardiovascular disease [5]. In stage two, characterized by the maternal syndrome, the leakage of debris and placenta-derived vesicles from the fetoplacental unit to the maternal circulation contributes to the systemic inflammation, endothelial damage, and dysfunction that are central in PE [5,7,8,9,10].

There are increases in proinflammatory cytokines as well as calprotectin, an inflammatory marker commonly used in the clinic, in the maternal circulation in PE [11,12,13]. Calprotectin is a heterodimer protein that is mainly expressed in myeloid cells, functioning as a promotor and amplifier of inflammation in various diseases [14]. It binds to pattern recognition receptor toll-like receptor 4 (TLR4) on the cell surfaces of monocytes, activating the innate immune system [15]. TLR4 also recognizes lipopolysaccharide (LPS) from the outer surface membranes of Gram-negative bacteria. A significant increase in serum calprotectin levels has been described in the third trimester of PE pregnancies [16].

There is increasing evidence that dysbiosis of the gut microbiome and increased intestinal permeability are involved in several diseases that often involve low-grade inflammation, such as obesity, diabetes, and hypertension [17,18,19,20]. The gut microbiome plays important roles in host metabolism, immune responses, intestinal barrier function, and intestinal permeability [21,22]. Different bacterial strains can convey different effects in the host, for example, through differences in short-chain fatty acid (SCFA) production or the amount of LPS on the cell surface [19,23,24,25]. It is thought that LPS translocation over an impaired gut–blood barrier is a contributor to systemic inflammation, with several other effects to follow, such as increased insulin resistance [17,18,19]. The use of probiotics has often shown beneficial effects on these parameters [21,26,27].

The fungal component of the gut flora, the mycobiome, is far less studied. Fungi make up around 0.1% of the gut microbiome [28], but as a fungal cell is >100 times larger than a bacterial cell, fungi still make up a substantial part of the microbiome biomass [29]. The gut mycobiome is known to play a role in modulating immune responses both locally and systemically in the host [30]. Decreased mycobiome family biodiversity has been shown in obesity [31], and mycobiome dysbiosis has been shown in IBD [32,33]. However, to the best of our knowledge, the gut mycobiota has not been studied in PE.

During a normal pregnancy, a shift in the gut microbiome composition is seen, including a decrease in diversity and an increase in the abundance of the phyla *Proteobacteria* and *Actinobacteria* [34]. *Proteobacteria* are often associated with inflammation in non-pregnancy but might play a more beneficial role in pregnancy [35]. These changes are accompanied by metabolic changes that are beneficial and necessary for a normal pregnancy, such as increased fat accumulation in the first trimester and increased insulin resistance in the third trimester [36]. However, in obese pregnant women and pregnancies complicated by PE, studies have shown differential gut microbiome compositions [23,37,38,39,40,41,42]. One study has shown a causal relationship between dysbiosis and PE through bacterial translocation in a mouse model that received fecal microbiota transplantation (FMT) from PE patients [42]. Other studies indicated that gut dysbiosis leads to blood pressure elevation and PE development, mainly through the effects of dysregulated plasminogen activator inhibitor-1 (PAI-1), butyrate, and other SCFAs [42,43,44]. A recent study showed that a lower abundance of the butyrate-producing genus *Coprococcus*, as well as lower serum butyrate levels, can already be detected at 28 gestational weeks in obese women who later develop late-onset PE [45]. The MoBa study in Norway has shown that the consumption of milk-based probiotics lowers the risk of developing PE [46,47].

Although studies have shown an altered gut flora in PE patients, the number of studies is limited [39,40,41,42,44,48], and there might be differences among populations due to environmental differences. Studies in Scandinavian populations are scarce. Furthermore, the mycobiome has not yet been studied in PE or in normal pregnancy. Another aspect that has been overlooked in studies of the gut microbiome in PE is the presence of symptoms from the gastrointestinal tract, such as loose/watery stool and local inflammation in the intestine.

Studies on possible roles of the gut microbiome in PE contribute to new knowledge regarding the pathophysiology of a very common complication of pregnancy. They also provide potential new targets for prediction and treatment. Our hypothesis is that the damaged endothelium in the vascular bed causes an impaired gut–blood barrier, indirectly affecting the gut microbiota, and that these changes can cause or aggravate the inflammatory response both systemically and locally in the gut.

The purpose of this study was therefore to evaluate differences in both the bacterial and fungal components of the gut microbiome in women with PE compared to gestational age-matched controls in southern Sweden. This was performed using 16S and ITS rRNA gene sequencing for bacteria and fungi, respectively. In addition, the calprotectin levels were evaluated in maternal feces and blood as a marker of inflammation, and symptoms in the gastrointestinal tract were evaluated.

## 2. Materials and Methods

### 2.1. Ethics Statement

The study was approved by the Regional Ethics Committee at Lund University, Sweden (2017/419). All subjects gave their written, informed consent before entering the study. All samples were coded before analysis.

### 2.2. Definitions

PE was defined according to the ISSHP 2018 diagnostic criteria [2]: de novo hypertension (systolic blood pressure ≥140 mmHg or diastolic blood pressure ≥90 mmHg) manifesting after gestational week 20, combined with proteinuria or signs of organ dysfunction (thrombocytopenia, impaired liver function, progressive renal insufficiency, pulmonary edema, or new-onset cerebral of visual disturbances). Severe PE was defined as a systolic blood pressure ≥160 mmHg, a diastolic blood pressure ≥110 mmHg, or the prevalence of the organ dysfunctions mentioned above. Early-onset PE was defined as PE manifesting before 34 gestational weeks, and late-onset PE manifested after 34 gestational weeks.

### 2.3. Study Cohort and Questionnaire

Patients in the third trimester with newly diagnosed PE or normotensive control pregnancies were recruited for the study. Recruitment took place between 2017 and 2020 at delivery wards at Skåne University Hospital and at selected antenatal care centers in Malmö and Lund, Scania, Sweden. The exclusion criteria were type 1 or 2 diabetes, gestational diabetes in the current pregnancy, IBD, celiac disease, BMI > 30 before pregnancy, and treatment with antibiotics within the past 2 weeks.

A questionnaire on diet regarding the intake of probiotics and meat, medications, obstetric and medical history, and smoking habits as well as symptoms related to PE or the gastrointestinal tract was filled in by the patients. The questionnaire was designed to give an overview of factors that could be related to variations in the studied parameters and was partly based on questionnaires for the screening and monitoring of IBD and irritable bowel syndrome (IBS) patients. Demographic data and the results of a routine oral glucose tolerance test (OGTT) performed during gestational week 28 were acquired from medical records.

### 2.4. Sample Collection

Blood samples were drawn from the antecubital vein, and feces samples were collected in sterile 20 mL tubes at inclusion. The samples were immediately stored in a fridge for a maximum of 48 h. The blood samples were centrifuged, and plasma was stored at −80 °C until analysis. The feces samples were stored at −80 °C until further analysis.

### 2.5. Protein Extraction and Calprotectin ELISA

The calprotectin concentration in feces and plasma was determined using a two-site sandwich ELISA technique (Immundiagnostik AG, Bensheim, Germany). A total of 15 mg of feces from each patient sample was used for protein extraction according to manufacturer’s protocol. The absorbance was measured at 450 nm, and a curve was fitted to standard samples using a 4-parameter algorithm. Calprotectin concentrations were calculated from the resulting equations.

### 2.6. Reference Values

Feces calprotectin >50 mg/kg is considered borderline-positive, and >100 mg/kg is considered positive, according to manufacturer. Factors that can increase the levels of fecal calprotectin include the use of NSAIDs (non-steroidal anti-inflammatory drugs), intercurrent gastrointestinal infection, and malignancy. The manufacturer gave no reference value for plasma calprotectin since levels can vary depending on the preanalytical conditions. Variations in preanalytical conditions were kept to a minimum in our study.

### 2.7. DNA Extraction, 16S rRNA, and ITS1 Gene Amplicon Sequencing

Starting with the 54 enrolled women (29 C and 25 PE), 46 fecal samples (26 C and 20 PE) were collected and underwent total genomic DNA extraction. The taxonomic structure of the gut microbiome can be defined using DNA amplification and the sequencing of hypervariable regions in highly conserved sequences of the genome. For bacteria, hypervariable regions of the highly conserved 16S rRNA gene are used for this purpose. The standard marker used in fungal taxonomy is the internal transcribed spacer (ITS) region of the rRNA gene. The generated sequences are clustered to generate operational taxonomic units (OTUs), which can be compared with reference libraries to define the taxonomic structure at the species level.

Total gDNA was extracted from the fecal samples using the DNeasy PowerSoil Pro kit (QIAGEN, Venlo, The Netherlands) and subsequently quantified with fluorometric quantification using the Invitrogen Qubit Fluorometer assay (Thermofisher, Waltham, MA, USA). Specific primers were used to target the hypervariable V3–V4 regions of the 16S gene (341f: 5′-CCTACGGGNGGCWGCAG-3′ and 805r: 5′-GACTACNVGGGTWTCTAATCC-3′) [49] and the ITS1 region (ITS1f: 5′-CTTGGTCATTTAGAGGAAGTAA-3′ and ITS2r: 5′-GCTGCGTTCTTCATCGATGC-3′) [49], respectively, for the bacterial and fungal microbiota, and were fitted with overhang Illumina adapters. Amplicon library preparation was conducted according to Illumina protocols [50,51]. Paired-end sequencing was carried out using a 600-cycle MiSeq™ Reagent Kit v3 and an Illumina MiSeq system (Illumina, San Diego, CA, USA) at the Biology Department of Florence University, Italy.

### 2.8. Sequence Data Processing

The DADA2 pipeline (version 1.14.1) was used to reconstruct amplicon sequence variants (ASVs) from the fastq files. Sequence variant reconstruction and statistical analyses were performed in the R environment (version 3.4.3). Amplification primers were detected and removed from the Illumina raw data using cutadapt (version 1.15). Low-quality reads were discarded using the filterAndTrim function. A minimum length of 50 bp was set to avoid technical constructs that could be created during the amplification step. The DADA2 function was used to perform denoising after error rate remodeling (learnErrors function). Forward and reverse reads were merged using the mergePairs function. Chimeric sequences were removed using the removeBimeraDenovo function. A taxonomic classification was assigned to the sequence variants using DECIPHER Bioconductor package version 2.14.0. For ITS sequencing, the reference database used was the RDP classifier Warcup ITS training dataset. The ASV table output sequences were analyzed using the phyloseq R package.

### 2.9. Statistical Analyses

SPSS Statistics 27 (IBM) was used for Mann–Whitney U-tests to compare patient demographics, the levels of calprotectin in plasma and feces, and oral glucose tolerance test (OGTT) values between PE and normotensive groups. Kruskal–Wallis tests were performed to compare questionnaire answers regarding smoking, gastrointestinal tract symptoms, diet, and probiotics consumption.

For microbiota analyses, R software and the vegan package (version 2.5-6) were used. Alpha diversity was analyzed using a one-way analysis of variance (ANOVA), and beta diversity was analyzed using the Bray–Curtis dissimilarity index using the vegdist function. Differences in sample composition were analyzed using a principal coordinate analysis (PCoA) based on vegdist among all samples using the cmdscale R function. The effects of diet and PE on variances in microbiota composition among samples were studied using a permutational multivariate analysis of variance (PERMANOVA, adonis2 function of the vegan package with 1000 permutations). The effects of gestational age and BMI on variances in microbiota composition were analyzed using an environmental fitting using the R function envfit with 1000 permutations. A *t*-test was performed to investigate the difference between two distinguishable groups in the analysis. DESeq2 was used to estimate fold changes in the gut mycobiota between normotensive pregnancy and PE. Differential abundances in the bacterial microbiota between groups were analyzed using a linear discriminant analysis effect size (LEfSe) analysis, with LDA scores = 3.

Results with *p* values < 0.05 were considered significant.

## 3. Results

### 3.1. Patient Characteristics and Questionnaire

A total of 54 women were included in the study. In total, five individuals from the PE group and three individuals from the control group failed to leave feces samples, and blood samples were not collected from one individual from the control group. One individual from the PE group was excluded from calculations regarding patient characteristics since they had a duplex pregnancy. The final inclusions were 25 PE patients (20 feces samples and 25 blood samples) and 29 women with normotensive pregnancies, designated as controls (26 feces samples and 29 blood samples).

The patient demographics for singleton pregnancies are summarized in Table 1. The groups were similar with regards to age, pre-pregnancy BMI, gestational age at inclusion, and fetal sex ratio. There were more nulliparous women in the PE group. Gestational age at birth and birth weight were significantly lower in the PE group. The control group and PE group had similar smoking habits and dietary habits regarding the consumption of meat; other foods of animal origin; and probiotic foods such as fermented milk, yoghurt, kombucha, miso, tempeh, and other foods or supplements containing probiotics.

The results from the questionnaire regarding gastrointestinal symptoms showed that patients with PE had significantly more frequent bowel movements and a tendency towards more loose/watery stool than the controls (*p* = 0.024 and *p* = 0.059, respectively) (Table S1).

### 3.2. Oral Glucose Tolerance Test at Gestational Week 28

The Mann–Whitney U-test showed a significant difference in the 2 h glucose levels during the oral glucose tolerance test (OGTT) at 28 weeks, with lower levels in the group that later developed PE (*p* = 0.030). For the controls (n = 28) the median blood glucose level was 6.65 mmol/L (IQR: 6.13–7.15 mmol/L), while the median level in the PE group (n = 21) was 6.10 mmol/L (IQR: 5.10–6.70 mmol/L) (Table 1).

### 3.3. Calprotectin Levels in Plasma and Feces

**Figure 1:**
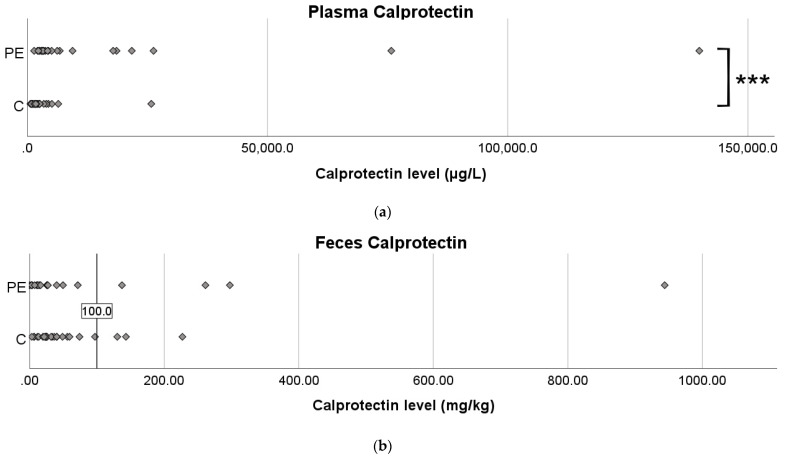
Plasma (**a**) and feces (**b**) calprotectin levels in controls vs. PE. (**a**) PE: N = 25, median 3764 µg/L, IQR 2897–13,639 µg/L; C: N = 28, median 1819 µg/L, IQR 1176–2612 µg/L. (**b**) PE: N = 20, median 21.0 mg/kg, IQR 8.4–66.2 mg/kg; C: N = 26, median 29.1 mg/kg, IQR 21.2–56.9 mg/kg. The line indicates the upper normal reference level for fecal calprotectin. Mann–Whitney U-test for significance, *** *p* < 0.001.

A Mann–Whitney U-test showed significantly higher plasma calprotectin levels in the PE group (N = 25, median 3764 µg/L, IQR 2897–13,639 µg/L) compared to controls (N = 28, median 1819 µg/L, IQR 1176–2612 µg/L) (*p* < 0.001), with high variation between subjects (Figure 1a). The difference remained significant (*p* < 0.001) after excluding outliers, defined as values greater than Q3 + 1.5*IQR for each group, which excluded two PE patients and three controls (Figure S1a). There was no significant difference in feces calprotectin levels (PE: N = 20, median 21.0 mg/kg, IQR 8.4–66.2 mg/kg; C: N = 26, median 29.1 mg/kg, IQR 21.2–56.9 mg/kg, *p* = 0.375) for the entire sample (Figure 1b) or after excluding outliers (Figure S1b).

### 3.4. Micro- and Mycobiomes Based on 16S and ITS rRNA Gene Sequencing

Using ITS1 and 16S rRNA gene amplicon sequencing, the fungal and bacterial communities were profiled, respectively, for 43 (24 C and 19 PE) and 42 (25 C and 17 PE) samples. Samples that generated insufficient library concentrations due to amplification problems were excluded (one PE patient and two controls for ITS1 amplification and three PE patients and one control for 16S rRNA amplification). The average number of reads obtained for each sample from MiSeq ITS sequencing was 157,543. After the Dada2 pipeline was used to construct ASVs, the average number of reads for each sample was 12,769. After removing adapters, filtering, and removing unknown ASVs, 345 known ASVs remained, representing around 10% of the total number of reads. The reduction in retained reads after all the ASV detection steps of the pipeline was approximately 90%. This result is explained by the fact that most fungal diversity is unknown, and ITS sequences in the International Nucleotide Sequence Database (INSD: GenBank, EMBL, and DDBJ) revealed that this region is not equally variable in all groups of fungi. The 16s rRNA sequencing generated reads with a quality compatible with downstream analysis for 39 samples (24 C and 15 PE). The ITS1 sequencing was successful for all 43 amplicon libraries. All metagenome sequences were deposited in the European Nucleotide Archive under Bioproject PRJEB59212.

### 3.5. Diversity Indices

**Figure 2:**
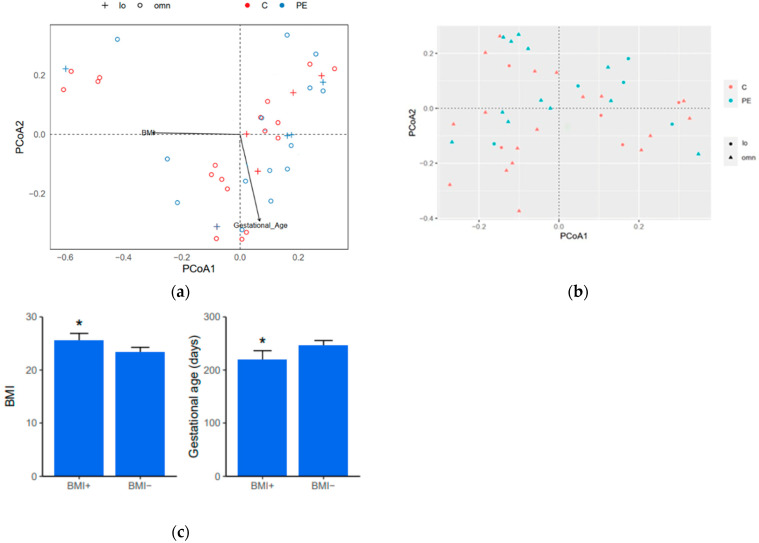
Diversity indices for fungal and bacterial microbiome composition in control and PE pregnancies, based on ITS and 16S sequencing. Beta diversity illustrated with principal coordinate analysis (PCoA) for fungal (**a**) and bacterial microbiota (**b**). Vectors indicate increasing BMI and gestational age. (**c**) The two bar plots quantitatively depict how BMI and gestational age influence the differences between the two groups BMI+ and BMI-, separated in the PCoA plot for fungal microbiota (**a**). C: control, PE: preeclampsia, l.o: lacto-ovo vegetarian, omn: omnivorous, veg: vegan diet. *t*-test for significance. * *p* = 0.0361.

Between-sample dissimilarities (i.e., beta diversity) were evaluated using a classical multidimensional scaling (MDS) of a data matrix, also known as a principal coordinate analysis (PCoA) on a quantitative index (Bray–Curtis). The beta diversity across fungal and bacterial communities in control and PE pregnancies, visualized in PCoA plots, did not show any significant differences between the groups (Figure 2a,b). The alpha diversity in the bacterial and fungal microbiomes, according to the Chao1 and Shannon diversity indices, did not differ between the groups.

However, six samples, including both controls and PE, were clustered distant from the other samples in the upper left quadrant in the fungal PCoA plot (Figure 2a). This group could not be distinguished in the 16S analysis. This group was renamed BMI+ since it was arranged in a position compatible with the direction of the BMI vector (Figure 2a). The remaining samples were included in a second group, renamed BMI-, where the samples were arranged opposite the BMI vector direction. A *t*-test showed that the BMI+ group had a significantly higher pre-pregnancy BMI (mean of 25.65 kg/m^2^ compared with 23.36 kg/m^2^, *p* = 0.0361) as well as a lower gestational age at inclusion (mean of 220 days compared with 246 days, *p* = 0.0411) (Figure 2c). An environmental fitting analysis showed that gestational age was positively correlated with PCoA1 and negatively correlated with PCoA2, with an R-squared value of 0.326 (*p* = 0.002). BMI was negatively correlated with PCoA1, but the R-squared value was only 0.098 (*p* = 0.192).

The R-squared values obtained with a PERMANOVA support that neither diet nor PE explain the variations in the bacterial microbiome composition (R-squared = 0.050 and 0.031, respectively) or mycobiome composition (R-squared = 0.036 and 0.018, respectively).

### 3.6. Gut Bacterial Microbiome Composition

**Figure 3:**
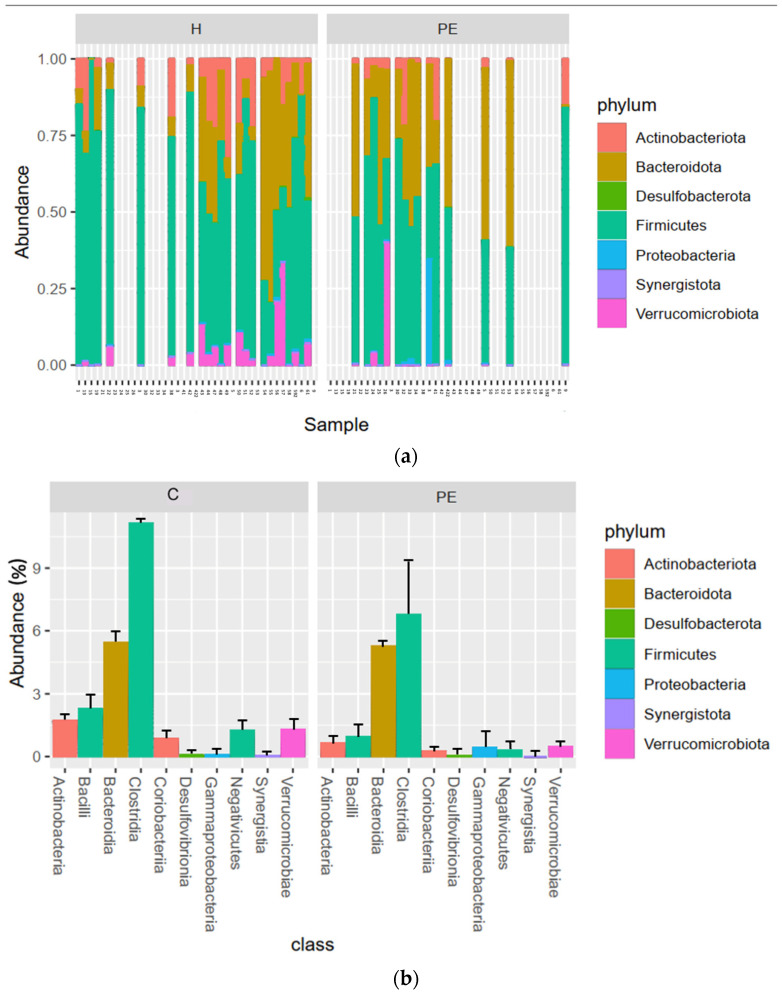
Stacked bar plots of the relative abundance with the standard deviation of bacterial taxa in the gut microbiota in 24 control and 15 PE pregnancies, based on successfully sequenced samples of the 16S rRNA gene. Abundances at the phylum level in individual samples (**a**). Mean abundances according to group at the class level, with colors indicating phyla (**b**). Cutoff for taxon inclusion = 0.005%.

**Figure 4:**
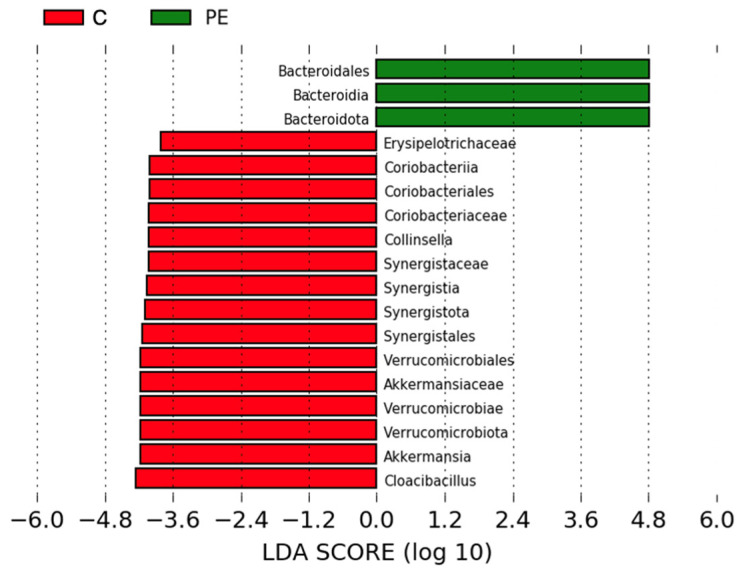
Linear discriminant analysis effect size (LEfSe) analysis showing significantly differential relative abundances of bacteria in control and PE pregnancies at all taxonomic levels. LDA score threshold = 3. Green = higher in PE, red = higher in controls. Results with *p* values < 0.05 were considered significant and are shown in the figure.

At the phylum level, we saw that *Firmicutes* were dominant in most patients, followed by *Bacteroidetes* (Figure 3a). The relative abundance of *Firmicutes* was lower in PE, especially the class *Clostridia*, although this was not significant (*p* > 0.05) (Figure 3b). The differential abundances in the bacterial microbiota between groups were analyzed using a linear discriminant analysis effect size (LEfSe) analysis, and the significantly differentially abundant taxa are summarized in Figure 4.

This large effect size with a high *p*-value (>0.05%) is due to the high variability of the *Clostridia* class in the PE samples. Random sampling error is more likely to produce substantial differences between groups, even when no effect exists in the population.

*Verrucomicrobia* were present in most patients at varying, mostly low levels, while *Desulfobacteria* and *Synergistia* were present only at low levels. *Proteobacteria* also displayed a very low relative abundance in all patients except for one PE patient, where they constituted around 35%. The relative abundance of the class *Actinobacteria* was lower in the PE group (Figure 3b), being present in only one PE patient but in all controls, although the difference was not significant.

An LEfSe analysis showed 18 significantly differentially abundant taxa in PE (Figure 4). At the phylum level, *Bacteroidetes* were enriched and *Verrucomicrobia* and *Synergistia* were depleted. At the class level, *Bacteroidia* were enriched and *Coriobacteriia*, *Synergistia*, and *Verrucomicrobiae* were depleted. At the order level, *Bacteroidales* were enriched and *Coriobacteriales*, *Synergistales*, and *Verrucomicrobiales* were depleted. At the family level, *Erysipelotrichaceae*, *Coriobacteriaceae*, *Synergistaceae*, and *Akkermansiaceae* were depleted. At the genus level, *Collinsella*, *Akkermansia*, and *Cloacibacillus* were depleted. *Coriobacteriia*, *Coriobacteriales*, *Coriobacteriaceae*, and *Collinsella* belong to the phylum *Actinobacteria*.

### 3.7. Gut Mycobiome Composition

The average number of reads obtained for each sample from MiSeq sequencing was 157.543, and after the Dada2 pipeline was used to construct ASVs, the average number of reads for each sample was 12.769. The reduction in retained reads after all the amplicon sequence variant detection steps of the pipeline was approximately of 90%.

This result is explained by the fact that most fungal diversity is unknown, and ITS sequences in the International Nucleotide Sequence Database (INSD: GenBank, EMBL, and DDBJ) revealed that this region is not equally variable in all groups of fungi. For this reason, we did not perform any stratification according to groups but instead show the intestinal mycobiota of all samples, characterizing the mycobiota of pregnant women.

**Figure 5:**
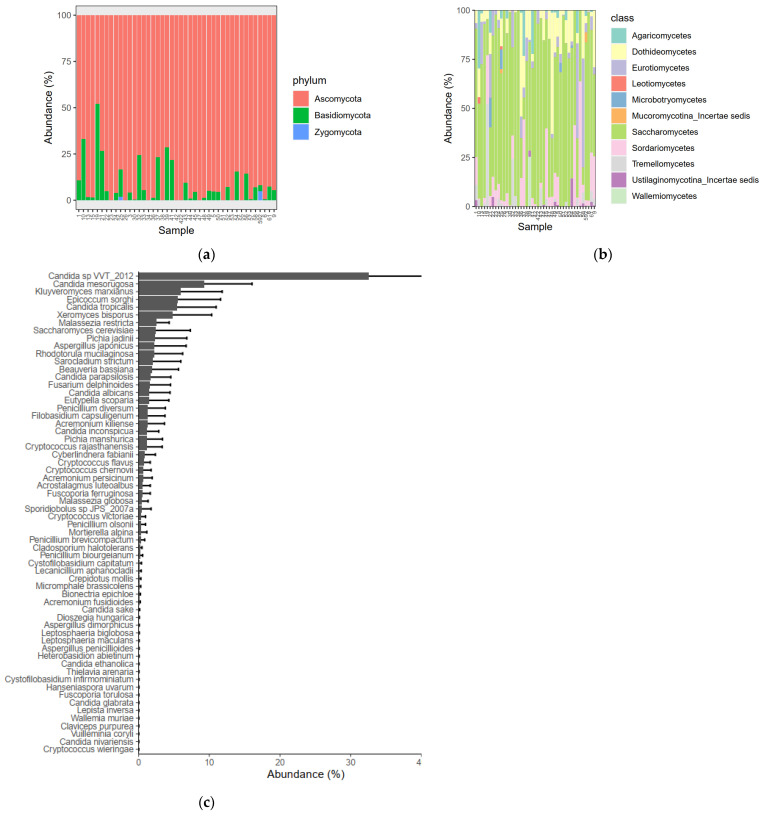
Bar and stacked bar plots showing relative percentage abundances of mycobiome taxonomic bins in ITS gene sequence datasets: the phylum (**a**) and class levels (**b**) are shown individually, while the species level is shown as means across the cohort (**c**). The most abundant fungal species are displayed. Cutoff for taxon inclusion = 0.01%.

The relative abundances of the most common fungal phyla across all included individuals showed the dominance of *Ascomycota*, which constituted >50% of the community in all samples except one, where *Basidiomycetes* constituted 55% (Figure 5a). *Basidiomycetes* were present, with a mean abundance of 8.4% in 67.4% of the samples. The third detected phylum, *Zygomycota*, was observed in only two samples (one PE and one C patient), where it constituted minimal traces (<5%).

Subsequently, we tried to reach a deeper taxonomic level of fungal investigation, descending from class to the species level. We reconstructed a stacked area graph using the ggplot2 R package with the classes of fungi and their corresponding relative abundances (Figure 5b).

At the class level, the most abundant class, with a mean relative abundance of 50%, was the *Saccharomycetes* class, followed by the *Dothideomycetes* and *Sordariomycetes* classes, present in 70% of the samples at a mean abundance of 14% each, all belonging to the *Ascomycota* phylum (Figure 5b). A graphic examination of the relative abundances at the class level confirmed the results from the diversity analysis, showing no differences in fungal gut microbial profiles between PE and controls. The average abundances of the most abundant fungal species across all samples showed that *Candida* species were most abundant, reaching a relative abundance of 35% with the species *Candida sp VVT_2012*. However, other species such as *Candida mesorugosa*, *Candida tropicalis*, *Candida parapsilosis*, and *Candida albicans*, albeit in a decreasing order of relative abundance, were important colonizers of the microbiomes of pregnant women (Figure 5c).

The only significant fold change that was calculated between control and PE was for ASV-36 (corresponding to the genus *Peniophora*), which was less represented in PE patients than in controls.

## 4. Discussion

In this study we report significantly differential abundance in 18 bacterial taxa in PE, including enrichment at the phylum level of *Bacteroidetes*, and depletion of *Verrucomicrobia* and *Synergistia*. Significant depletion at the genus level included *Akkermansia* and *Cloacibacillus*. We report increased plasma levels of calprotectin and a higher frequency of gastrointestinal symptoms in PE patients. Furthermore, we report a first description of the gut mycobiome in pregnancy, indicating correlations with gestational length and BMI. Our results suggest that gut dysbiosis might play a role in the pathophysiology of PE and that gastrointestinal symptoms might be a previously overlooked manifestation of the maternal syndrome and a potential target for therapy.

We are studying an association between a disease and microbial profiles, but everything is complicated by the simultaneous state of pregnancy, for which a change in the intestinal bacterial microbiome has already been described.

Physiologically, gut the microbiota of pregnant women is dominated by two major bacterial phyla, *Firmicutes* and *Bacteroidetes*, followed by *Actinobacteria*, *Proteobacteria*, and *Verrucomicrobia*. This compositional pattern of bacterial phyla is generally seen in the human gut microbiota and is widely observed in normal non-pregnant adult populations, despite the occasional perturbance of low-abundance phyla. Healthy pregnant women exhibit a consistent enterotype that is clearly driven by the abundance of several dominant genera, such as *Bacteroides*, *Prevotella*, and *Ruminococcus*. This enterotype composition has various fluctuations across all gestational stages, with a slight reduction in the *Ruminococcus* enterotype in the last stage of pregnancy. Four genera, *Ruminococcus*, *Collinsella*, *Megamonas*, and unclassified *Erysipelotrichaceae*, increase continuously with gestational age, whereas *Ruminococcus*, *Dialister*, and unclassified *Lachnospiraceae* decrease continuously [52].

Similar to earlier studies on other populations, we observed significant differences in the gut flora composition in PE patients from the south of Sweden. In line with other studies [40,41], we observed a significant increase in *Bacteroidetes* in PE. Wang et al. also showed increased blood and feces levels of LPS in PE [41]. As a major contributor to LPS biosynthesis, increased *Bacteroidetes* abundance can increase inflammation through increased expression of TNF and IL-6, which are known to be elevated in PE through TLR4 [53]. Injections of LPS in pregnant rodents have been shown to cause PE-like symptoms [54,55].

The genus *Akkermansia*, belonging to the phylum *Verrucomicrobia*, was significantly depleted in PE, in line with other studies [40,42]. *Akkermansia* is almost the only genus in the phylum *Verrucomicrobia*, both of which appeared to be significantly reduced in the LEfSe analysis. This may define it as the most representative genus of the *Verrucomicrobia* phylum depletion in PE. The significant changes at different taxonomic levels in the LEfSe analysis likely also included similar overlaps in some of the other taxa. *Akkermansia* is involved in gut permeability homeostasis, based on its mucin-degrading ability [56,57], and *Akkermansia muciniphila* is associated with a low risk of diabetes [58], obesity [59], and high levels of inflammation [60]. At least one study indicated increased gut permeability in PE using the gut permeability marker zonulin [61]. Moreover, supplementation with *A. muciniphila* is protective against several cardiometabolic features [62]. It is possible that the depletion of *Akkermansia* in PE patients is coupled with the pathophysiology through increased gut permeability and inflammation.

The genus *Cloacibacillus*, belonging to the phylum *Synergistia*, was also significantly depleted in PE. As it is mucin-degrading and SCFA-producing [63,64], *Cloacibacillus* depletion might have negative effects on intestinal permeability and immune regulation.

The increased frequency of loose/watery stool observed in PE could be associated with the decrease in *A. muciniphila* and increase in *Bacteroidetes*. Earlier studies have shown that stool consistency is negatively correlated with species richness and positively correlated with the *Bacteroidetes*/*Firmicutes* ratio and that *A. muciniphila* abundance is positively correlated with colon transit time [65].

A tendency towards depletion was seen in the class *Actinobacteria* in PE, which was present in only one PE patient but all controls. Although we did not perform strain analyses, it is reasonable to believe that the probiotic strain *Bifidobacteria* made up part of the *Actinobacteria* depletion, being the largest strain in this class. *Bifidobacteria* are known for their anti-inflammatory properties, which result from Treg stimulation, increased IL-10 secretion, and the regulation of T helper cell mediated inflammatory responses [66,67,68]. Depletion of the family *Bifidobacteriaceae* and the genus *Bifidobacteria* in PE was indicated in a newly published study [48]. A skewed immune homeostasis because of *Bifidobacteria* depletion might play a role in the systemic inflammatory response seen in PE.

To the best of our knowledge, this study is the first to provide a description of the gut mycobiome in PE. According to our results, gestational age explains 32.62% of the variation in gut mycobiome composition in the entire cohort. The gut mycobiome is known to play immunomodulatory roles both locally and systemically [30]. The observed differences in mycobiome diversity correlating with BMI and gestational length suggest that the fungal flora, like the bacterial flora, exerts immunomodulatory effects during normal pregnancy. *Zygomycota*, which were observed in minimal traces in only one PE and one C patient, are known to be under-represented in obesity. Family biodiversity has also been shown to be lower in obesity [31]. Metabolic changes in normal pregnancy are, in some areas, similar to those of obesity, including reduced insulin sensitivity in late pregnancy [36].

Our results did not allow us to show any difference in the overall mycobiome diversity in PE compared to controls due to a high rate of retained reads after all amplicon sequence variant detection steps of the pipeline. For this reason, we limited ourselves to charting the fungal profile of all samples of pregnant women. Although there was a significant depletion of the *Peniophora* genus in PE, it is impossible to ascertain if there was a true difference since so many ASVs were unmeasurable or absent in the samples. The amount of unmeasurable or absent ASVs was related to known difficulties in ITS sequencing, e.g., the fact that the gut mycobiome constitutes only 0.1% of the total microbiome [28]. Moreover, Nilsson and colleagues estimated that more than 10% of the fully identified fungal ITS sequences are incorrectly annotated at the species level [69]. Fungal communities still represent a poorly studied “black box” for the human gut microbiome, and therefore many individuals do not have a valid classification. Furthermore, it is known that a large number of commensal gut fungi are an environmental signature, rather than commensals whose alteration reflects a difference in the human physiology in health and disease. It is likely that the fungal microbiome is more influenced by environmental factors than by human-physiology-related factors.

In line with other studies [11,12,13], we report a significant increase in calprotectin plasma levels in PE, which was expected, considering the systemic inflammatory response in PE. However, we could not see any differences in the fecal levels of calprotectin, indicating that there might not be any significant local inflammatory response in the gastrointestinal tract per se. Some individuals (three controls and four PE patients) displayed high values, and a high level of variation was seen between subjects, especially in the PE group. The majority of patients displaying high values had early-onset PE. It is possible that subgroups within PE display gastrointestinal involvement as a feature. However, high feces calprotectin did not correlate with more frequent bowel movements or loose/watery stool.

Although the blood glucose levels obtained in the standard OGTT showed significantly lower values in patients who later developed PE, it is unclear whether this has any clinical relevance in general or for the gut microbiota specifically. All levels were within normal reference values. Diabetes mellitus is associated with gut dysbiosis [17,18,19]. High blood glucose, or gestational diabetes mellitus, is a risk factor for PE [2]. However, there are no studies indicating that lower levels would also be a risk factor. The patients with the earliest onset of PE manifested before they underwent the OGTT, and therefore we do not have values from these patients.

The role played by the gut microbiome in PE and pregnancy in general is still poorly understood, and further studies are needed. This study provides a glimpse into the gut mycobiome in pregnancy, indicating that there might be composition changes during pregnancy. There is also a need for a more in-depth analysis with targeted metagenomics to determine the strain-specific changes in taxa that are differentially abundant in PE.

In summary, we suggest that increased *Bacteroidetes* and depleted *Synergistia*, *Akkermansia*, and possibly *Actinobacteria* might play a role in PE pathophysiology. This might be mediated through the combined effects of decreased gut membrane permeability, increased LPS signaling, decreased SCFA production, decreased Treg activity, and an increased T1 inflammatory response. The gut mycobiome might also be an important player in immunomodulatory changes in pregnancy. However, in this cohort the mycobiome was not altered in PE. Interestingly, the data also suggest that increased bowel movements and loose/watery stool might be a part of the maternal syndrome of PE, something that clinicians should pay more attention to in the clinical management of PE.

## Figures and Tables

**Table 1:** Patient demographics, questionnaire answers (smoking, diet, and probiotic food consumption), and OGTT results for singleton pregnancies included in the present study ^1^.

Parameter	Controls (C)	Preeclampsia (PE)	*p*-Value
N =	29	24	
Age (years)	31 (28–34.5)	30.5 (28–33.75)	0.632
Pre-pregnancy BMI (kg/m^2^)	23.7 (22.1–24.9)	22.7 (20.8–25.0) ^2^	0.233
Nulliparous (%)	65.50%	87.5%	0.055
Gestational age at inclusion (weeks + days)	36 + 1 (32 + 1–40 + 0)	35 + 0 (32 + 4–37 + 0)	0.221
Gestational age at birth (weeks + days)	40 + 1 (39 + 0–41 + 1)	36 + 0 (33 + 5–37 + 2)	<0.001 ***
Birth weight (g)	3680 (3252–4012)	2490 (1850–2796)	<0.001 ***
Sex of fetus (% boys/% girls)	55.2%/44.8%	58.3%/41.7%	0.819
Smoker (% no/% yes/% quit before pregnancy)	93.1%/0%/6.9%	87.0%/4.3%/8.7% ^3^	0.514
Diet (% vegan/% lacto-ovo vegetarian/% omnivorous)	3.4%/20.7%/75.9%	0%/20.8%/79.2 %	0.731
Probiotic food items consumed > 1 time/week	2 (1.5–2)	2 (2–2)	0.327
Gestational week 28 OGTT 2 h plasma glucose (mmol/L)	6.65 (6.13–7.15)	6.10 (5.10–6.70) ^4^	0.030 *

^1^ Numbers presented as medians (interquartile ranges, Q1–Q3). The Mann–Whitney U-test was used for significance for all parameters except smoking, diet, and probiotic consumption, where the Kruskal–Wallis test was used. * *p* <0.05, *** *p* < 0.001. ^2^ n (C) = 28, n (PE) = 23; ^3^ n (PE) = 23, ^4^ n (C) = 28, n (PE) = 21, including one duplex PE pregnancy. *: *p* ≤ 0.05.

## Data Availability

Raw sequencing data were deposited in the European Nucleotide Archive (ENA) under the accession code PRJEB59212.

## Supplementary Materials

The following supporting information can be downloaded at https://www.mdpi.com/article/10.3390/biom13020346/s1, Table S1: Summary of questionnaire answers regarding gastrointestinal symptoms; Figure S1: Plasma and feces calprotectin levels after excluding outliers.
