# Supplementary material for: Gut Micro- and Mycobiota in Preeclampsia: Bacterial Composition Differences Suggest Role in Pathophysiology
Source: Biomolecules. 2023 Feb 10;13(2):346. doi: 10.3390/biom13020346 (PMC9953204; doi:10.3390/biom13020346)
Supplement: Supplementary file 1 [file biomolecules-13-00346-s001.zip › biomolecules-2046821-supplementary.pdf]

Supplementary Material

Table S1. Summary of questionnaire answers regarding gastrointestinal symptoms.[1]

Question	Answer	C (N (%))	PE (N (%))	*P*-value
1. After gestational week (g.w.) 20: How often do you have bowel movements?	< 3 times/week	2 (6.9%)	0	**0.024 ***
	3–5 times/week	11 (37.9%)	6 (26.1%)	
	1 time/day	13 (44.8%)	8 (34.8%)	
	>1 time/day	3 (10.3%)	9 (39.1%)	
Total:		N = 29	N = 23	
2. After g.w. 20: How often is the stool loose/watery?	Never or rarely	20 (69.0%)	8 (36.4%)	0.059
	Around 25% of the time	2 (6.9%)	7 (31.8%)	
	Around 50% of the time	5 (17.2%)	4 (18.2%)	
	Around 75% of the time	2 (6.9%)	3 (13.6%)	
	Always, 100% of the time	0	0	
Total:		N = 29	N = 22	
3. After g.w. 20: How often is the stool hard?	Never or rarely	9 (31.0%)	7 (29.2%)	0.315
	Around 25% of the time	4 (13.8%)	9 (37.5%)	
	Around 50% of the time	6 (20.7%)	3 (12.5%)	
	Around 75% of the time	8 (27.6%)	5 (20.8%)	
	Always, 100% of the time	2 (6.9%)	0	
Total:		N = 29	N = 24	
4. After g.w. 20: How often have you noticed blood in the stool?	Never or rarely	29 (100%)	23 (95.8%)	0.272
	Sometimes	0	1 (4.2%)	
	Often	0	0	
	Most of the time	0	0	
	Always	0	0	
Total:		N = 29	N = 24	
5. After g.w. 20: How often have you noticed that the stool was black?	Never or rarely	14 (48.3%)	15 (62.5%)	0.414
	Sometimes	7 (24.1%)	3 (12.5%)	
	Often	5 (17.2%)	4 (16.7%)	
	Most of the time	3 (10.3%)	2 (8.3%)	
	Always	0	0	
Total:		N = 29	N = 24	
6. After g.w. 20: How often have you noticed mucus in the stool?	Never or rarely	23 (82.1%)	21 (91.3%)	0.486
	Sometimes	4 (14.3%)	1 (4.3%)	
	Often	1 (3.6%)	1 (4.3%)	
	Most of the time	0	0	
	Always	0	0	
Total:		N = 28	N = 23	
7. After g.w. 20: How often do you experience nausea, acid reflux or vomiting?	Never or rarely	8 (27.6%)	8 (34.8%)	0.748
	< 1 day/month	0	0	
	1 day/month	0	0	
	2–3 days/month	4 (13.8%)	3 (13.0%)	
	1 day/week	10 (34.5%)	4 (17.4%)	
	>1 day/week	5 (17.2%)	5 (21.7%)	
	Every day	2 (6.9%)	3 (13.0%)	
Total:		N = 29	N = 23	

	Never or rarely	15 (51.7%)	14 (58.3%)	0.799

1

8. After g.w. 20: How often do you experience abdominal pain?	< 1 day/month	2 (6.9%)	2 (8.3%)	
	1 day/month	2 (6.9%)	0	
	2–3 days/month	3 (10.3%)	2 (8.3%)	
	1 day/week	3 (10.3%)	2 (8.3%)	
	>1 day/week	4 (13.8%)	3 (12.5%)	
	Every day	0	1 (4.2%)	
Total:		N = 29	N = 24	

[1] Kruskal–Wallis test for significance. * *p*-value < 0.05

(**a**)

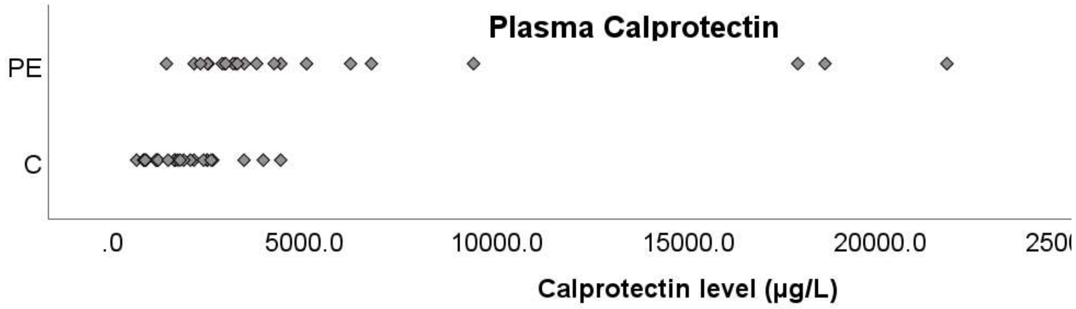

(**b**)

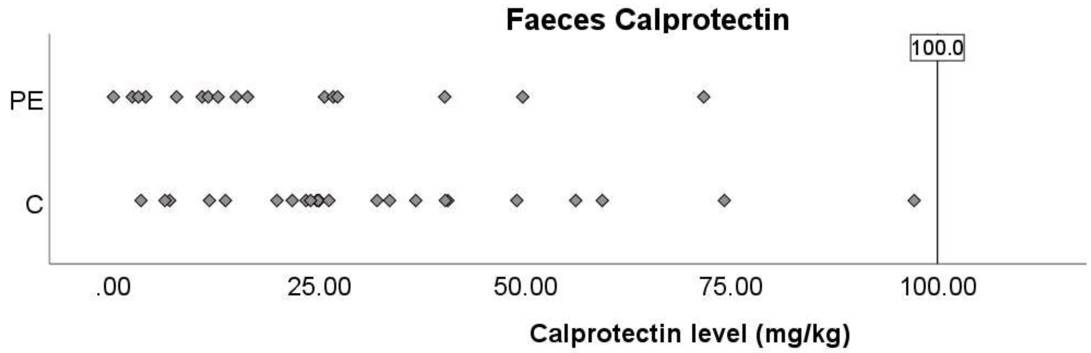

Figure S1. Plasma (a) and feces (b) calprotectin levels in controls vs. PE after excluding outliers, defined as values greater than Q3 + 1,5*IQR for each group. a) PE: N = 23, median 3443 µg/L, IQR 2867–6755 µg/L; C: N = 25, median 1711 µg/L, IQR 1157–2475 µg/L. b) PE: N = 17, median 14.9 mg/kg, IQR 5.8–33.7 mg/kg; C: N = 23, median 25.0 mg/kg, IQR 19.8-40.6 mg/kg. The line indicates the upper normal reference level for feces calprotectin. Mann–Whitney U-test for significance, *** $p < 0.001$.
